# Incidence of Otolaryngological Symptoms in Patients with Temporomandibular Joint Dysfunctions

**DOI:** 10.1155/2014/824684

**Published:** 2014-06-24

**Authors:** E. Ferendiuk, K. Zajdel, M. Pihut

**Affiliations:** ^1^Department of Dental Prosthetics, Consulting Room of Functional Disorders of Masticatory Organ, Jagiellonian University Medical College, 4 Montelupich Street, 31-155 Cracow, Poland; ^2^Department of Otolaryngology, Jagiellonian University Medical College, 2 Śniadeckich Street, 31-531 Cracow, Poland

## Abstract

The functional disorders of the masticatory organ are the third stomatological disease to be considered a populational disease due to its chronicity and widespread prevalence. Otolaryngological symptoms are a less common group of dysfunction symptoms, including sudden hearing impairment or loss, ear plugging sensation and earache, sore and burning throat, difficulties in swallowing, tinnitus, and vertigo. The diagnostic and therapeutic problems encountered in patients with the functional disorders of the masticatory organ triggered our interest in conducting retrospective studies with the objective of assessing the incidence of otolaryngological symptoms in patients subjected to prosthetic treatment of the functional disorders of masticatory organ on the basis of the analysis of medical documentation containing data collected in medical interviews.* Material and Methods*. Retrospective study was conducted by analyzing the results of medical interviews of 1208 patients, who had reported for prosthetic treatment at the Functional Disorders Clinic of the Department of Dental Prosthetics of Jagiellonian University Medical College in Cracow between 2008 and March 14, 2014.* Results*. Otolaryngological symptoms were observed in 141 patients. The most common symptoms in the study group were earache and sudden hearing impairment; no cases of sudden hearing loss were experienced.

## 1. Introduction

Temporomandibular joint dysfunctions are the third stomatological disease to be considered as a populational disease due to its chronicity and widespread prevalence. The disorders occur in more than 10% of population, restricting individuals' capability to perform everyday functions at home and work. According to epidemiological data, the number of patients with the painful form of the disorders is increasing (to about 40%) while the age of patients with temporomandibular disorders is being lowered. The problem is more common in females than in males [1].

Functional disorders of the masticatory organs are often manifested by acute or chronic pain symptoms in the temporomandibular joints and/or masseter muscles. Impaired dynamics of mandibular movements is manifested as restricted or enhanced range of jaw openings, deviations in the course of abduction and adduction of the mandible, and lack of symmetry of mandibular lateral movements. Acoustic symptoms within the joints, manifested as popping and cracking sounds, are an evidence of the lack of coordination between the articular head temporomandibular joint articular discs during mandible movements [2]. Otolaryngological symptoms are a less common group of dysfunction symptoms, including sudden hearing impairment or loss, ear plugging sensation and earache, sore and burning throat, difficulties in swallowing, tinnitus, and vertigo [3–5]. Due to the latter symptom, patients may experience fear when moving around. Tinnitus is acoustic sensations heard in one or both ears or inside the patient's head when no acoustic stimuli are coming from the outside. Tinnitus may be experienced as squeaks, whistles, chirps, bubbling sounds, pulsations, howls, paper rustle, or sea humming [6].

Facial pains and headaches are one of the symptoms of the painful form of the functional dysfunction of the masticatory organ, commonly misdiagnosed and treated as pain of some other etiology [7–9]. The impact of the emotional factors in the development of temporomandibular joint dysfunction and common concomitance of otolaryngological symptoms should also be noted. Tinnitus, chronic facial pains, and dysfunctions were commonly reported in depressive patients [9–11].

Other symptoms of the dysfunction include disorders of the visual organ, neck and shoulder girdle muscles, spinal muscles, and even upper limbs and chest muscles. In such cases, differential diagnostics is the key element of the diagnostics and treatment process; however, it is not an easy task [12, 13].

Subjective hearing organ symptoms that accompany the functional disorders of the masticatory organ are broadly reported in the literature [14–18]. The incidence of these symptoms is in the range of about 5 to 30% for earache and about 30% for tinnitus [19–22]. The reported causes for otolaryngological symptoms include common embryonic origin of the ear and the masseter muscles and the compression of vessels, nerves, and ligaments by posteriorly translocated articular heads of the mandible in the middle and inner ear regions [3, 4]. This is due to missing teeth not being replaced, particularly in the support zones, as well as to pathological teeth attrition, promoting the reduction in occlusal height and posterior translocation of articular heads in central occlusion [23, 24].

Occlusal parafunctions are responsible for the hyperactivity of masticatory muscles, including lateral superior pterygoid muscles, with one of the attachments located within the articular disc. Contractures of these muscles occurring upon pathological motor habits translocate the disc towards the front and lead to posterior location of mandibular heads. Many authors report a relationship between otolaryngological symptoms, particularly earache, and masticatory muscle disorders [8, 11, 16, 25].

Patients reporting for prosthetic treatment had often undergone otolaryngological consultations previously, with results suggesting that the complaints were of an origin other than being located within the middle or inner ear. Another much smaller group of patients with otolaryngological disorders are patients reporting for prosthetic treatment of otolaryngological symptoms constituting an indication of specialist consultation. In most cases, the results of such consultations are negative [19, 24].

The diagnostic and therapeutic problems encountered in patients with the temporomandibular joint dysfunction triggered our interest in conducting retrospective studies with the* objective* of assessing the incidence of otolaryngological symptoms in patients subjected to prosthetic treatment of the functional disorders of masticatory organ on the basis of the analysis of medical documentation containing data collected in medical interviews.

## 2. Material and Methods

Retrospective study was conducted by analyzing the results of medical interviews of 1208 patients, both males and females, aged 19 to 50 years, who had reported for prosthetic treatment at the Consulting Room of Temporomandibular Joint Dysfunction, Department of Dental Prosthetics of the Jagiellonian University Medical College in Cracow between 2008 and March 14, 2014. Each patient was subjected to specialist examination of the masticatory organ function. The first part of the examination consisted of medical interview. Based on the specialist diagnostics including the assessment of the range and symmetry of mandibular movements and pain upon palpation of temporomandibular joints and masseter muscles, patients were diagnosed with dysfunctions of the stomatognathic system. The following symptoms were taken into consideration when analyzing the results of medical interviews: sudden hearing impairment or loss, earache or sore throat, burning throat, tinnitus (squeaks, swishes, bubbling sounds, pulsations, whistles, chirps, and sea humming), and vertigo. The number of otolaryngological symptoms in individual patients and the total number of all symptoms in the study group were also assessed. The duration of the incidence of otolaryngological symptoms ranged from 3 weeks to 14 months before the commencement of prosthetic treatment, and the reported otolaryngological complaints were experienced every day. No history of otolaryngological procedures within the three preceding years was reported in the medical interview.

## 3. Results

Retrospective analysis was conducted on 1208 case report forms from 952 women (78.8% of all patients) and 256 men (21.2% of all patients) treated between 2008 and March 14, 2014, in the Laboratory of Functional Disorders of the Masticatory Organ of the Department of Dental Prosthetics of the Jagiellonian University Medical College in Cracow. Medical documentation included the results of medical interviews containing information on the reason for patient's reporting for the treatment, subjective symptoms, duration of symptoms, intensity of symptoms, and precious or subsequent otolaryngological verification of the reported complaints. It should be mentioned that examination results in patients undergoing otolaryngological consultation prior to reporting for prosthetic treatment were negative.

The symptom most commonly reported in medical interviews was earache, experienced by 69 out of 1208 subjects. In 58 cases it was a one-sided located pain. In 21 cases, the pain was an acute, persistent pain lasting for several hours during the day; in the remaining patients, the intensity of pain was considered mild. In seven cases, the earache was accompanied by the sensation of compression and fullness in the ear. No increase in body temperature or intensification of pain in horizontal position, usually accompanying acute or chronic otitis, was observed. In addition, of symptoms manifesting within the ear region, 14 cases of hearing impairment were reported, while none of the assessed patients experienced sudden hearing loss. The second most common otolaryngological symptom was tinnitus, experienced by 45 and manifesting as squeaks (in 21 patients), sea humming (11 patients), whistling (8 patients), and ticking sounds (5 patients). In 26 patients tinnitus was unilateral whereas in the rest of the respondents it was bilateral. In 4 cases, a sensation of the presence of a foreign body (worm) within the ear canal was reported. Patients reported high persistence of tinnitus that interfered with their normal occupational and social activities; each of the patients highlighted that the sensations were perceived as louder and more troublesome during the night's rest periods. In four patients, tinnitus was accompanied by the ear plugging sensation, thus leading to concomitance of 2 otolaryngological symptoms in the same patient. Other otolaryngological symptoms included in the analysis were sore throat, reported by 4 patients, and vertigo, reported by 9 patients. Vertigo was described as the sensation of whirling, swaying, and instability of patients' surroundings. In 4 patients, vertigo was accompanied by nausea. All patients reporting vertigo upon medical investigation were previously consulted by otolaryngologists, with otolaryngological treatment being initiated for this reason in some of them, not leading to resolution of the complaints.

**Figure 1:**
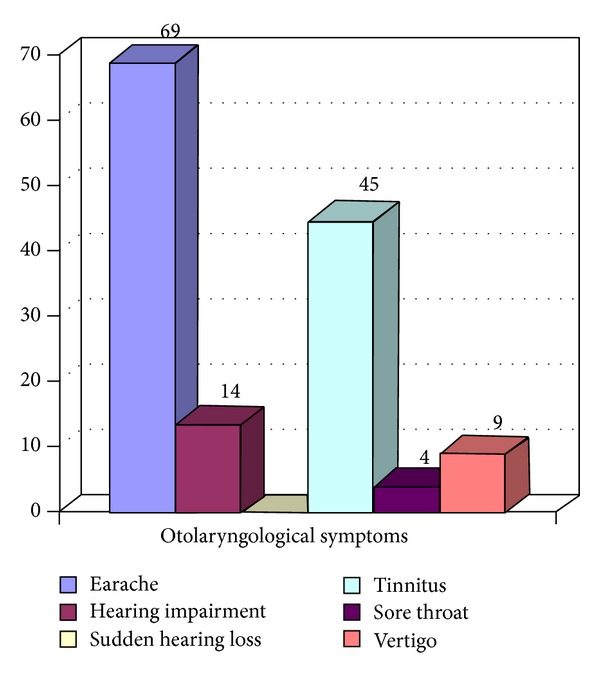
Graphic presentation of the incidence of individual otolaryngological symptoms in the study group.

The results of the analysis of otolaryngological symptoms in the group of patients undergoing prosthetic treatment for functional disorders of the masticatory organ are presented in Table 1, while graphical representation of these results is provided in Figure 1.

## 4. Discussion

Otolaryngological symptoms accompanying pathological posterior translocation of the mandible were first observed in 1920 by Monson who reported a case of sudden hearing loss due to abnormal location of mandibular heads [26]. Five years later, Decker described a series of hearing loss and impairment cases in patients with deep occlusion and posterior location of mandibular heads within the temporomandibular joints. In 1933, Goodfriend observed the relationship between the incidence of tinnitus and dysfunction of the stomatognathic system [22, 24]. It is wrongly assumed that the first physician to associate the hearing disorders with masticatory organ dysfunction was Costen. He published his observations as late as in 1934, and therefore he was the fourth researcher who paid attention to the concomitance of tinnitus, vertigo, and earaches with complaints related to the stomatognathic system [27]. As highlighted by several authors, common phylo- and ontogenetic development of masseter muscles, facial muscles, and ear muscles (tensor palati and tensor tympani muscles), originating from the first pharyngeal arches, is not unimportant for the concomitance of otolaryngological symptoms and masticatory organ dysfunction. In addition, posteriorly translocated mandibular head (due to missing teeth, pathological teeth attrition, or trauma) may compress the tympanic artery and vein, leading to blood supply disorders within the middle ear and constituting an important cause of hearing disorders. At the same time, compression by the articular head may damage the tympanic cord, leading to contracture of the stapedius muscle in a reflex mechanism transmitted via the facial nerve. In addition, the course of the auriculotemporal nerve in the temporomandibular joint region promotes its compression by the mandibular head, generating an impulse for reflexive contracture of the tensor tympani muscle and leading to hearing impairment or tinnitus symptoms. Anatomical fissures between the articular cavity and the middle ear, such as petrotympanic or petrosquamous fissures, are routes for transmission of inflammatory infections. Another possible reason for the concomitance of both types of symptoms is the transmission of excess mechanical forces by the discomalleolar ligament or direct compression on the auriculotemporal nerve [3, 4].

The analysis of the obtained results suggests that otolaryngological symptoms are important element of the symptomatology of the functional disorders of the masticatory apparatus, particularly in cases of negative results of consultations pertaining to the condition of the middle or inner ear or tinnitus. Ear-related symptoms may accompany neoplastic growths in the region (Reichert syndrome, i.e., tympanic neuralgia accompanying glossal tumors) or may be due to injuries or contusions of face or head [13].

The fact that tinnitus may be a symptom of other diseases, not related to the stomatognathic system, such as otosclerosis, Ménière's disease, tumors within the cerebellopontine angle, and acoustic neuroma, should be taken into account in differential diagnostics [6].

It is difficult to identify the leading causal relationship theory for the symptoms in question; in difficult cases of functional disorders accompanied by auriculovestibular symptoms, numerous possible causes for the dysfunction of the middle and the inner ear as well as the functional disorders of the masticatory organ should be taken into account [22, 24].

## 5. Summary

In cases when otolaryngological origin of symptoms such as earache or sore throat, hearing impairment, tinnitus, or vertigo is excluded, functional disorders of the masticatory organ should be taken into consideration [1, 4, 5, 9, 10, 14–25, 27].

## Figures and Tables

**Table 1:** Incidence of individual otolaryngological symptoms reported in medical interviews.

The number of analyzed interviews	1208 patients					
Otolaryngological symptom	Earache	Hearing impairment	Sudden hearing loss	Tinnitus	Sore/burning throat	Vertigo
Number of symptoms reported in medical interviews	69	14	0	45	4	9
Skip on the amount of the tested-‰	5.71‰	1.16‰	0	3.72‰	0.33‰	0.75‰
